# The Comparative Study of Serum Estrogen and Lipid Profile in Pre- and Post-menopausal Women as Atherosclerosis Risk Factors in Pakistan

**DOI:** 10.7759/cureus.65604

**Published:** 2024-07-28

**Authors:** Anum Chaudhry, Khazina Ikram, Kaneez Ayesha, Mehrish Waheed, Noor Ulain, Amna Tariq, Tooba Khalid

**Affiliations:** 1 Faculty of Rehabilitation and Allied Health Sciences, Riphah International University, Islamabad, PAK; 2 Faculty of Rehabilitation and Allied Health Sciences, Riphah International University, Islamabad, Pakistan, Islamabad, PAK

**Keywords:** hdl, atherosclerosis, plasma lipids, estrogen, menopause

## Abstract

Background: Menopause signifies the eternal termination of menstruation in women as a consequence of ovarian action loss, typically occurring around the age of 51 years. Cardiovascular disease is the leading cause of death among post-menopausal women, which may be due to lower levels of estrogen and lipid profile. The present study was undertaken to evaluate serum estrogen and lipid profile status to assess the risk of atherosclerosis in both pre- and post-menopausal women.

Objectives: The objective of this study is to explore the relationship between estrogen and lipid levels of women in pre- and post-menopausal stages.

Methodology: A comparative cross-sectional study was conducted at Railway General Hospital Rawalpindi. A total of 100 participants were included of which 50 were pre-menopausal and 50 were post-menopausal women. Laboratory examination and questionnaires from the study population were used for data collection. Through the enzymatic method, serum cholesterol, triglycerides, low-density lipoprotein, and high-density lipoprotein (HDL) were assessed. Serum very low-density lipoprotein (VLDL) levels were calculated via Friedwald’s components VLDL=TG/5.0. An enzyme-linked immunosorbent assay kit was used for estrogen measurement. For statistical analysis, Student's t-test and the Pearson correlation test were used.

Results: Women after menopause have significantly high serum cholesterol, low-density lipoproteins, VLDLs, and triglycerides while HDL-c levels were significantly low (*P*<0.001). Levels of estrogen were low in post-menopausal females (*P*<0.001) as compared to menstruating women. Estrogen with HDL concentrations showed a positive correlation with an r value of 0.08556 while LDL levels showed a negative correlation with a r value of -0.26219.

Conclusion: This comparative study explores the relationship between estrogen and lipid levels in pre- and post-menopausal women. Low estrogen with changed lipid variables was observed. Decreased cardiovascular protective HDL-c marks that menopause is a phase that acts as an independent risk factor for atherosclerosis.

## Introduction

Menopause, as defined by the World Health Organization (WHO), signifies the eternal termination of menstruation in women as a consequence of ovarian action loss, typically occurring around the age of 47-51 years [1]. Menopause is identified retrospectively without menstruation, marking the last menstrual period. Menstruation generally begins during puberty and persists until menopause interrupted only during pregnancy [2]. Women today can expect to live approximately one-third of their lives after menopause. Following the termination of menstruation, the female ovaries are not able to synthesize substantial volumes of estrogen, leading to the symptoms and health issues associated with low levels of estrogen [3].

Alterations in the metabolism of the lipid profile occur in women after menopause as a result of the various hormonal changes. Women after menopause face variations in insulin metabolism, clotting mechanisms, fibrinolysis, and impairment of vascular endothelium functioning [4]. In post-menopausal women, the occurrence of heart diseases is partially caused due to changes in the lipids panel during the menopausal evolution. Excess low-density lipoproteins (LDLs) have been evidenced in the involvement of coronary heart disease [5]. Experiments carried out in the 20th century showed cholesterol as the primary risk factor for atherosclerosis. Then research conducted on the characterization of lipoprotein practices led to the concept of insudation of lipids as a factor in the development of atherosclerosis, with LDL being the foremost threat aspect in the expansion of cardiovascular diseases (CVDs) [6]. Coronary artery disease (CAD) is the primary reason for death in women after menopause. Elder aged women after menopause are considered 4 to 8 times more likely to die due to CAD compared to any other ailment [7]. The findings of Framingham's research evidenced the matter that in females after 45 years of age the levels of CAD morbidity increase rapidly compared to the morbidity ratio in males of that particular phase. Numerous aspects as risk factors have been stated which are accountable for advancement in atherosclerosis [4].

Due to estrogen, the vessel's permeability is enhanced by improved production of the nitrous oxide, it sustains the well-balanced panel of lipoproteins. These estrogens enrich the antioxidant properties by alleviation of the endothelial cells and alteration of fibrinolytic protein [8]. Women mislaid all these cardiac defending utilities after menopause. Women after menopause bear the highest risk of developing heart diseases. The estrogen dearth is the key feature contributing to the disruption of plasma lipid panels in females after post-menopausal which is linked to higher CVD risk [9]. Estrogen replacement therapy has lowered cardiovascular threat by 25-50% in postmenopausal females by maintaining their lipid profiles [10].

Numerous factors contribute to the risk of CVDs; therefore, studies on endogenous hormones enhance our prediction of CVDs [11]. Regardless of the comprehensive investigations that linked estrogens and progesterone to the enhanced metabolism of lipids panel, the following factor is still ambiguous these alterations in steroid meditations are connected to lipid changes. CAD is obviously multi-factorial, so the facts regarding these endogenous hormones help in enhancing the calculation of atherosclerosis [12].

In women, the treatments related to the replacement of estrogen after menopause have declined the danger of heart disease by 25-50% by improving the lipid profile. Early intervention and prevention of dyslipidemia can decrease the peril of cardiac issues in elder aged women [13]. Our research study is designed to inaugurate variances of lipid status in pre- and post-menopausal women and it aims to evaluate the connection between the status of menopause and its related deviations in plasma profiles of lipids by serum estrogen level in both groups.

## Materials and methods

Study design and study population

A cross-sectional study was conducted on healthy women who were in the pre- and post-menopausal phase between September 2023 and January 2024 at Rawalpindi Railway General Hospital. The research ethics committee reviewed the protocol of the study and permitted it. A total of 100 participants were taken of which 50 were pre-menopausal women and 50 were post-menopausal women. Women who had experienced amenorrhea for 12 consecutive months without the use of any hormone therapy were selected as post-menopause and menstruating women with a healthy reproductive system without any disease history were selected as pre-menopause. Menstruating women above age 25 and post-menopausal women below age 70 were included in the study. All women with a history of any illness and disorder like CVD, hypertension, DM, cancer, hormonal therapies, or dyslipidemia drugs and women with factors like smoking, alcohol, sedentary behavior, and proficient sportspersons were excluded from this study.

Covariates

Demographic data was recorded from the study population. It included age, weight, height, menopause status, and health status of all women. Women with any disease history were noted.

Biochemical measurements

After overnight fasting of the study population, samples of whole blood were drawn and stored. Then serum samples were separated by centrifugation for further examination. For measurement of the lipid profile, the serum samples were loaded into the Selectra model Pro M Lite machine which was based on the phenomena of selective absorption of light by a substance. Through the enzymatic method serum cholesterol, triglycerides, high-density lipoprotein (HDL), and LDL were evaluated then serum VLDL was calculated via the usage of Friedwald’s components VLDL=TG/5.0 [14]. An enzyme-linked immune sorbent assay (ELISA) kit was used for measuring estradiol, which is based on the competitive binding immunoassay technique for quantitative investigations. Body mass index was calculated depending on the pre- and post-menopausal woman’s height and weight.

Statistical analysis

Descriptive statistics including the mean and standard deviation of both groups were calculated for parameters (i.e. age, BMI, estrogen, and lipid profile). For statistically evaluating the data, an independent Student t-test was used. P values <0.05 were considered statistically significant. For measuring the strength of the linear relationship between two variables, the Pearson test of correlation was calculated and r values were interpreted as positive or negative correlation.

## Results

**Figure 1:**
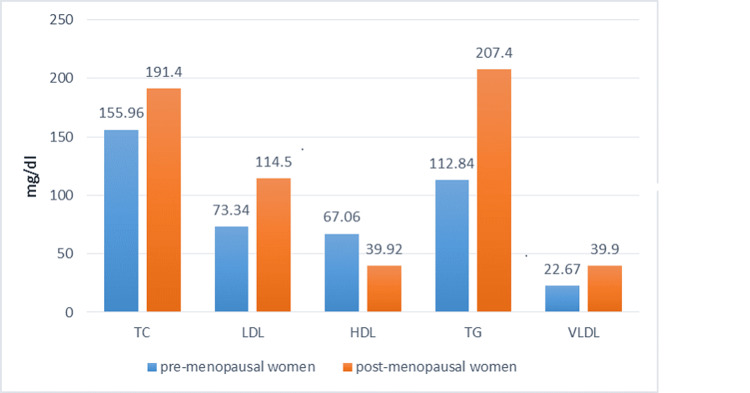
Comparison of the Lipid Profile in Pre- and Post-menopausal Women TC: Total cholesterol, LDL: Low-density lipoproteins, HDL: High-density lipoproteins, VLDL: Very low-density lipoproteins, TG: Triglycerides

**Figure 2:**
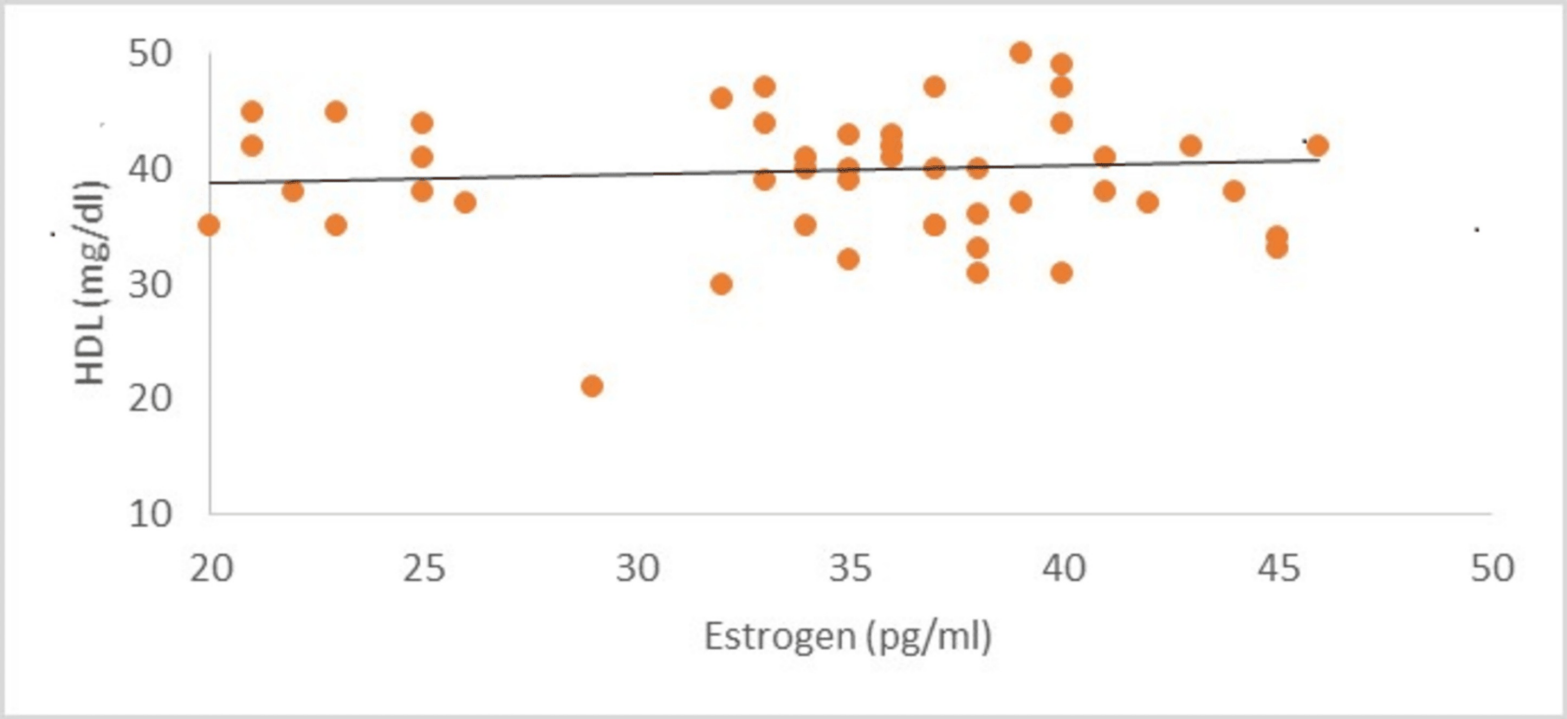
Correlation of Estrogen with HDL in Post-menopausal Women HDL: High-density lipoprotein

**Figure 3:**
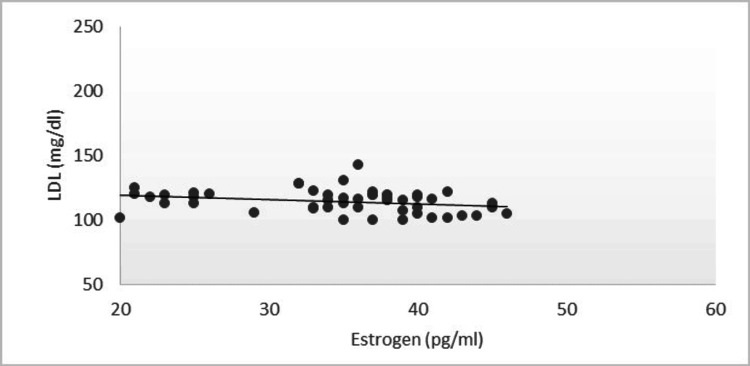
Correlation of Estrogen With LDL in Post-menopausal Women LDL: Low-density lipoprotein

In the present study, women after menopause have significantly high concentrations of serum cholesterol, triglycerides, and low- and very low-density lipoproteins as compared to women having menstruation (P<0.001) (Table 1). Post-menopausal women had a low concentration of high-density lipoproteins (P<0.001) (Figure 1). Levels of hormone estrogen were lower in post-menopausal women (P<0.001) as compared to menstruating women. According to the results of the present research, the average BMI of both categories of women was not significant statistically (P>0.05) (Table 2). Estrogen levels with HDL concentration showed a positive correlation with an r value of 0.08556 (Figure 2) while LDL levels showed a negative correlation with an r value of -0.26219 (Figure 3).

**Table 1:** Study of Parameters in Pre- and Post-menopausal Women n: Number of subjects; SD: Standard deviation; LDL: Low-density lipoproteins; HDL: High-density lipoproteins; VLDL: Very low-density lipoproteins

Parameters	Pre-menopausal Women (n=50) Mean (SD)	Post-menopausal Women (n=50) Mean (SD)	P-value
Cholesterol	155.96 (9.86)	191.4 (7.91)	˂0.001
LDL	73.34 (9.02)	114.5 (8.87)	˂0.001
HDL	67.06 (7.60)	39.92 (6.53)	˂0.001
Triglyceride	112.84 (8.79)	207.4 (7.64)	˂0.001
VLDL	22.67 (3.14)	39.90 (7.39)	˂0.001

**Table 2:** Age, BMI, and Serum Estrogen in Pre- and Post-menopausal Women n: Number of subjects; SD: Standard deviation; BMI: Body mass index

Parameters	Pre-menopausal Women (n=50) Mean (SD)	Post-menopausal Women (n=50) Mean (SD)	P-value
Age	30.52 (5.45)	57.54 (6.32)	˂0.001
BMI	21.95 (2.11)	21.95 (2.11)	0.993
Estrogen	272.96 (10.93)	34.78 (6.94)	˂0.001

## Discussion

Later to menopause, female ovaries lack sufficient production of estrogen and this reduction fallouts in the instability of the lipoprotein profile, opposing alterations in insulin and lipid metabolism, fibrinolysis, coagulation, and dysfunctional vascular endothelium [7]. Along with this, estrogen accomplishes numerous cardio-protective contrivances that can alter vascular tenor by amassed production of nitrous oxide. These estrogens enrich antioxidant properties by alleviation of the endothelial cells and alteration of proteins fibrinolysis. These all properties are cardiac defensive that women mislaid with the inception of menstruation [15]. In post-menopausal women, heart diseases, one of the foremost reasons for death, cause almost 53% of all deaths in females over this age [16]. Post-menopausal estrogen insufficiency, dyslipoproteinemia, and progressive age are significant risk factors for atherosclerosis and these risk factors are proven to be connected with increased progression of coronary artery disease in women [17].

In this recent research, serum cholesterol, triglycerides, LDL, and VLDL were significantly high in post-menstruating women compared to the pre-menopausal females of the study population (P <0.001). These findings align with those of Fatima et al., where total cholesterol is raised in post-menopausal women due to lower estrogen and (P< 0.001) is a statistically significant value [1]. Levels of HDL were observed low in women after menopause and it is interrelated by several other research studies of different regions, while research studies that controvert these results also exist [18]. Numerous findings by different researchers have validated that higher HDL concentrations are correlated with a lesser frequency of heart diseases [19]. Inversely, lower HDL is linked with the very high onset of coronary heart diseases. Different randomized have clearly revealed that exogenous estrogen lowers the LDL as well as elevates the HDL levels up to 10-15 % [20, 21]. 

In the present study, women after menopause had prominently higher values of serum LDL and the P-value is statistically significant (P <0.001). These research outcomes are consistent with a few other studies [4]. In our findings, post-menopausal females had raised triglycerides (P < 0.001) compared to the females before menopause. These findings are similar to the other research findings reported by Yeasmin et al. [22]. There is a greater release of fatty acids in blood circulation and increased accumulation of fats in post-menopausal women and these unnecessary free fatty acids offer a better substrate for liver triglyceride production [23]. 

Insufficient estrogen in women after menopause prompts the effective gathering of VLDL elements with cholesterol esters. These leftovers are greatly proficient in interaction with smooth arterial muscle cells [24]. These VLDLs are well recognized as alone constituent danger aspects for cardiovascular diseases. Concerning these risk factors that are impacting the female’s lipid panel will improve the cardiovascular risk of women after menopause [25].

In this study, all the features that could change lipid metabolism are omitted. BMI in our study population does not specify any imperative difference (P>0.05) and these results are aligned with a study carried out in India by Warjukar et al. [4]. So we have settled the fact that deficient serum estrogen is the reason for these fluctuations in females after menopause and is not interrelated to higher body mass index.

There are some limitations in this study. The study was restricted to a single hospital environment and there were limited resources. The time frame was limited and the sample size was small which might have impacted the depth of research. Diet and lifestyle factors were not catered for our study as diet also impacts lipid profile. More studies can be conducted to provide a more efficient understanding of the fact considering lifestyle and other factors that may influence the study parameters.

## Conclusions

This comparative study explores the relationship between estrogen and lipid levels of women in pre- and post-menopausal stages. A low estrogen level and change in lipid variables were observed. Decreased cardiovascular protective HDL marks that menopause is a phase that acts as an independent risk factor for atherosclerosis. In this study, lipid alterations of post-menopausal females advocate that this age group of females is at bigger threat of heart problems presently. Accordingly, it is very compulsory to reassure females that they undertake screening tests for these non-standard lipid profiles. Detailed health training is desired to save women from emerging vascular diseases. Primary and appropriate identification and exclusion will surely lessen the rates of mortality and morbidity in this high-risk population like Pakistan.

## References

[1] Fatima Y, Ramesh S (2017). A comparative study of serum estrogen and lipid profile in premenopausal and post-menopausal women as atherosclerotic risk factors. Int J Clin Biochem Res.

[2] Anagnostis P, Lambrinoudaki I, Stevenson JC, Goulis DG (2022). Menopause-associated risk of cardiovascular disease. Endocr Connect.

[3] Li H, Sun R, Chen Q, Guo Q, Wang J, Lu L, Zhang Y (2021). Association between HDL-C levels and menopause: a meta-analysis. Hormones (Athens).

[4] Warjukar P, Jha RK, Kute P (2020). Study of lipid profile, estradiol for evaluation of cardiovascular risk in pre-and post-menopausal women. Int J Curr Res Rev.

[5] Mach F, Baigent C, Catapano AL (2020). 2019 ESC/EAS Guidelines for the management of dyslipidaemias: lipid modification to reduce cardiovascular risk. Eur Heart J.

[6] Šojat D, Marušić R, Ormanac K, Marczi S, Bačun T (2023). Lipid profile of postmenopausal women. Medica Jadertina.

[7] Lambrinoudaki I, Armeni E (2024). Understanding of and clinical approach to cardiometabolic transition at the menopause. Climacteric.

[8] Lira-Silva E, Del Valle Mondragón L, Pérez-Torres I (2023). Possible implication of estrogenic compounds on heart disease in menopausal women. Biomed Pharmacother.

[9] Qian C, Liu J, Liu H (2024). Targeting estrogen receptor signaling for treating heart failure. Heart Fail Rev.

[10] Kumari P, Bano M, Sahay GJ (2019). Effect of diet and body mass index on the serum lipid profile in healthy premenopausal, perimenopausal, and postmenopausal tribal women of India. Int J Med Sci Public Health.

[11] Reddy Kilim S, Chandala SR (2013). A comparative study of lipid profile and oestradiol in pre- and post-menopausal women. J Clin Diagn Res.

[12] Allameh F, Pourmand G, Bozorgi A, Nekuie S, Namdari F (2016). The association between androgenic hormone levels and the risk of developing coronary artery disease (CAD). Iran J Public Health.

[13] Chen Z, Wu C, Huang Z (2024). Association between estrogen replacement therapy and heart failure in postmenopausal women: a systematic review and meta-analysis. Prev Med.

[14] Friedewald WT, Levy RI, Fredrickson DS (1972). Estimation of the concentration of low-density lipoprotein cholesterol in plasma, without use of the preparative ultracentrifuge. Clin Chem.

[15] Honigberg MC, Zekavat SM, Aragam K (2019). Association of premature natural and surgical menopause with incident cardiovascular disease. JAMA.

[16] Manafa P, Aguiyi N, Onyenekwe CC, Chukwuma GO, Okeke CO, Ihim AC (2015). Comparative assessment of lipid profile in pre-menopausal and menopausal women in Nnewi Nigeria. Eur Sci J.

[17] Dam V, van der Schouw YT, Onland-Moret NC (2019). Association of menopausal characteristics and risk of coronary heart disease: a pan-European case-cohort analysis. Int J Epidemiol.

[18] Ariadi A, Jamsari J, Yanwirasti Y, Siregar MF, Yusrawati Y (2019). Correlation between estrogen levels with lipid profile in menopause women in West Sumatera. Open Access Maced J Med Sci.

[19] Shenoy R, Vernekar P (2015). Fasting lipid profile in pre-and postmenopausal women: a prospective study. Int J Sci Stud.

[20] Tall AR (2021). HDL in morbidity and mortality: a 40+ year perspective. Clin Chem.

[21] Madsen CM, Varbo A, Nordestgaard BG (2017). Extreme high high-density lipoprotein cholesterol is paradoxically associated with high mortality in men and women: two prospective cohort studies. Eur Heart J.

[22] Yeasmin N, Akhter QS, Mahmuda S, Nahar S, Rabbani R, Hasan M, Salehin M (2017). Effect of estrogen on serum total cholesterol and triglyceride levels in postmenopausal women. J Dhaka Med Coll.

[23] Kumar S, Shah C, Oommen E (2012). Study of cardiovascular risk factors In pre and postmenopausal women. Int J Pharma Sci Res.

[24] Newman CB (2023). Effects of endocrine disorders on lipids and lipoproteins. Best Pract Res Clin Endocrinol Metab.

[25] Anyigor-Ogah C, Onwe PE, Okike P, Njoba S, Ottah-Umahi G, Okorocha AE, Igwe JC (2015). Coronary artery disease and menopause: a consequence of adverse lipid changes. IOSR J Dent Med Sci.

